# Cell biology of *Zymoseptoria tritici*: Pathogen cell organization and wheat infection

**DOI:** 10.1016/j.fgb.2015.04.002

**Published:** 2015-06

**Authors:** Gero Steinberg

**Affiliations:** Biosciences, University of Exeter, Exeter EX4 4QD, UK

**Keywords:** STB, *Septoria tritici* blotch, μm, micrometers, MAPK, mitogen-activated protein kinase, Infection biology, Phenotypes, *Septoria tritici* blotch, *Mycosphaerella graminicola*

## Abstract

Cell biological research in the wheat pathogen *Zymoseptoria tritici* (formerly *Mycosphaerella graminicola*) has led to a good understanding of the histology of the infection process. Expression profiling and bioinformatic approaches, combined with molecular studies on signaling pathways, effectors and potential necrosis factors provides first insight into the complex interplay between the host and the pathogen. Cell biological studies will help to further our understanding of the infection strategy of the fungus. The cellular organization and intracellular dynamics of the fungus itself is largely unexplored. Insight into essential cellular processes within the pathogen will expand our knowledge of the basic biology of *Z. tritici*, thereby providing putative new anti-fungal targets.

## Highlights

• Cell biology of the infection begins to shed light on the host–pathogen interaction.
• The cell biology of the fungal pathogen is highly understudied.
• Intensified cell biology research promises new fungicide targets and will help mode-of-action studies.

## 1 Introduction

*Septoria tritici* blotch (STB) is one of the most devastating diseases of wheat (Dean et al., 2012; Gurr and Fones, 2015). Cell biological research on the causative agent, the ascomycete *Zymoseptoria tritici* (formerly *Mycosphaerella graminicola* or *Septoria tritici*), is focused mainly on the host–pathogen interface. This commentary aims to provide an overview of the current state of cell biological knowledge across the life history of the fungus *Z. tritici*. Hitherto, our knowledge of this notable crop pathogen has been largely restricted to its invasion strategy. In addition, this article summarizes the available knowledge on the organization and function of its cells. However, due to space constraints, several important aspects of research on *Z. tritici*, such as fungicide resistance, genome organization, evolution and population biology are only mentioned briefly here. The interested reader is referred to detailed overview articles elsewhere (Cools and Fraaije, 2013; Croll and McDonald, 2012; Orton et al., 2011; Stukenbrock and McDonald, 2008; Suffert et al., 2011; and articles in this special issue).

## 2 Cell biology of the pathogen

**Fig. 1:**
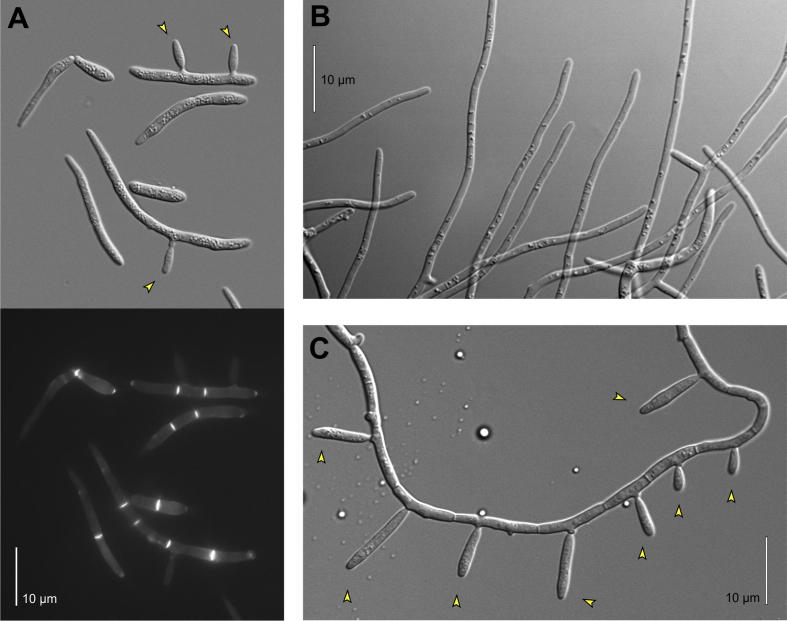
Growth forms of *Z. tritici*. (A) Pycnidiospores of strain IPO323, grown in yeast-extract/sucrose liquid medium at 18 °C. Spores are multi-cellular and form lateral (open arrowhead) and terminal buds (filled arrowhead). Scale bar represents 10 μm. (B) Hyphae of *Z. tritici*. Scale bar represents 10 μm. (C) Micropycnidia that are budding off from hyphal cells. Scale bar represents 10 μm.

Fungi show much morphological diversity. Different growth forms include single-celled yeasts, multi-cellular and tip growing hyphae and asexual and sexual spores (Charlile and Watkinson, 1995). The vegetative growth forms of *Z. tritici* fall into all three categories. The most common cell type, grown under laboratory conditions, is the macropycnidiospore (Fig. 1A). This form is often referred at the “yeast-like” stage. However, yeasts are uni-cellular, whereas macropycnidiospore are multi-cellular structures, consisting of 4–8 elongate cells (Fig. 1A, lower panel provides calcofluor staining of septa). The individual cells within this multi-cellular structure are ∼1.5–3.5 μm wide and can be up to ∼40–100 μm long (Sanderson et al., 1985; Wiese, 1987; note that data on dimensions varies between reports and that considerable morphological variation is seen in field isolates). However, *Z. tritici* also produces micropycnidiospores, which are small (∼1 μm wide, 5–10 μm long) and uni-cellular structures (Wiese, 1987; Eyal et al., 1987). They, therefore, fit the definition of a “yeast-like” growth form. These cells are formed by lateral budding from hyphae or macropycnidiospores (Fig. 1A, arrowheads; 1C, arrowheads). Neither macro- nor micropycnidiospores are dormant, are formed in asexual fruiting bodies (pycnidia) and are dispersed by rain splash (Sanderson et al., 1985). Finally, macropycnidiospores germinate to form thin hyphae, consisting of very elongated cells that extend by polar tip growth (Wiese, 1987; Fig. 1B). This morphogenic transition can be triggered in liquid culture upon nutrient deprivation and following an increase in temperature (Mehrabi et al., 2006b; Motteram et al., 2011). The ability to grow vegetatively in several growth forms is a characteristic of many pathogenic fungi, with hyphal growth being a prerequisite for invasion of host tissue (Gow, 1995).

**Fig. 2:**
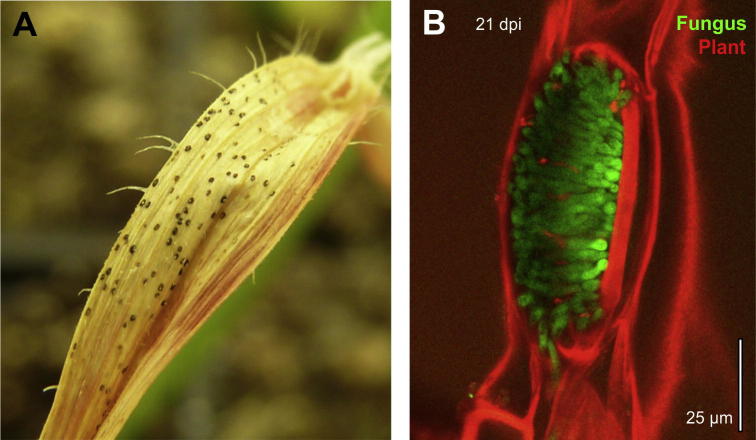
Pycnidia in *Z. tritici*. (A) Pycnidia on a necrotic wheat leaf. Image provided by N. Steinberg, Exeter, UK. (B) Confocal microscopy image of a pycnidium. Pycnidiospores (green), expressing green-fluorescent protein (Kilaru et al., 2015) fill the substomatal cavity. The plant cell wall is labelled with propidium iodide. Note the dense packing of pycnidiospores in the mature pycnidium, which corresponds well with published ultrastructural images (Mehrabi et al., 2006b). Image provided by Dr. H. Fones, Exeter, UK. Scale bar represents 25 μm.

Pycnidia appear as dark spots on the necrotic leaves (Fig. 2A), but can also be found in wheat stubble and debris (reviewed in Suffert et al. (2011)) where they were reported to oversummer on the surface of the soil (Hilu and Bever, 1957). Pycnidia develop underneath the stomata of infected wheat leaves and remain embedded in the plant epidermis (Weber, 1922, reviewed in Hilu and Bever (1957), Shipton et al. (1971), Kema et al. (1996a); Fig. 2B). They vary in size, usually ranging from 60 to 200 μm (Sanderson et al., 1985), depending on the fungal strain and on the density of the infection (Eyal and Brown, 1975; Kema and Annone, 1991), but also with stomata size variations of wheat cultivars (Rudd, pers. Comm.). *Z. tritici* also forms sexual ascospores, which have a role in over-seasoning (Eyal et al., 1987), are distributed by air and can spread the STB over hundreds of kilometers, whereas rain splashed macropycnidiospores dispersed more locally (Sanderson et al., 1985). Depending on the environmental conditions and agricultural practice, either asco- or pycnidiospores can be the primary source of inoculum in STB (Eriksen and Munk, 2003; Sanderson et al., 1985; reviewed in Suffert et al. (2011)). Ascospores are typically found in stubble and consist of 2 cells (Wiese, 1987). They are shorter (10–15 μm) and wider (2–3 μm) than the asexual macropycnidiospores. Ascospores are formed in perithecia, which are also sub-epidermal, 48–114 μm in diameter (Wiese, 1987) and appear after the pycnidia on infected leaves (Eriksen and Munk, 2003; Kema et al., 1996a; reviewed in Suffert et al. (2011)). Their formation depends on the meeting of strains of opposite mating types (Kema et al., 1996b) and, therefore, their appearance depends on the intensity of the epidemics (Cowger et al., 2002).

Insight into the regulation of morphology was provided recently. *Z. tritici* contains the mitogen-activated kinase (MAPK) ZtHog1 (named previously MgHog1). A *zthog1* deletion mutant showed abnormal spore morphology and multiple nuclei, suggesting a role of the MAPK signaling in pycnidiospore morphology (Mehrabi et al., 2006a). The morphological transition from “yeast-like” growth to hyphal growth was reported to be under the control of the MAPK and the cyclic AMP pathway (Mehrabi et al., 2006a,b, 2009) and the putative down-stream transcription factor ZtWor1 (Mirzadi Gohari et al., 2014). This transcription factor also regulates negatively hyphal fusion (anastomosis), which is found occasionally in early stages in infected leaf tissue, but which becomes very prominent in pycnidia formation (Hilu and Bever, 1957; see below).

*Z. tritici* carries 21 chromosomes (Goodwin et al., 2011). Eight of the smallest chromosomes appear to be non-essential (dispensable chromosomes) and are absent from some strains collected from the wild (Wittenberg et al., 2009). It was found that the dispensable chromosomes are lost frequently in meiosis (Wittenberg et al., 2009). The role of these dispensable chromosomes is not clear. In other pathogenic fungi, dispensable chromosomes carry genes involved in pathogenicity (Han et al., 2001; Hatta et al., 2002). The dispensable chromosomes in *Z. tritici* contain a high number of repetitive elements (Dhillon et al., 2014) and show a faster rate of evolutionary change (Stukenbrock et al., 2010). Thus, the dispensable chromosomes may facilitate rapid adaptation to changing environments (Wittenberg et al., 2009) or participate in development of fungicide resistance (Torriani et al., 2009). However, at present, there is no experimental evidence to support such proposed functions. Therefore, the biological role of dispensable chromosomes in *Z. tritici* remains elusive.

## 3 Cell biology of the infection process

From a cell biological point of view, the infection cycle of *Z. tritici* can be divided into the following phases: (1) entry of the fungus, (2) colonization of the plant tissue and (3) formation of fruiting bodies. Infection begins with the germination of either pycnidiospores or ascospores, both of which are pathogenic (Hilu and Bever, 1957; Eyal et al., 1987; Shipton et al., 1971; Suffert et al., 2011). They switch to hyphal growth upon contact with the leaf (Duncan and Howard, 2000; Kema et al., 1996a). This transition is essential for fungal infection and was first shown in a deletion mutant, lacking the mitogen-activated protein (MAP) kinase *Zthog1* (Mehrabi et al., 2006b) and a mutant defective in protein *N*-glycosylation (Δ*ztalg2*, Motteram et al., 2011; the protein was previously published as MgAlg2). Both mutant strains grow as pycnidiospores, but are unable to infect wheat plant tissue. As hyphal growth is a pre-requisite for tissue invasion in many fungal pathogens (Gow, 1995), these results suggest that a defect in morphogenic switching precludes plant infection in the mutant strains.

**Fig. 3:**
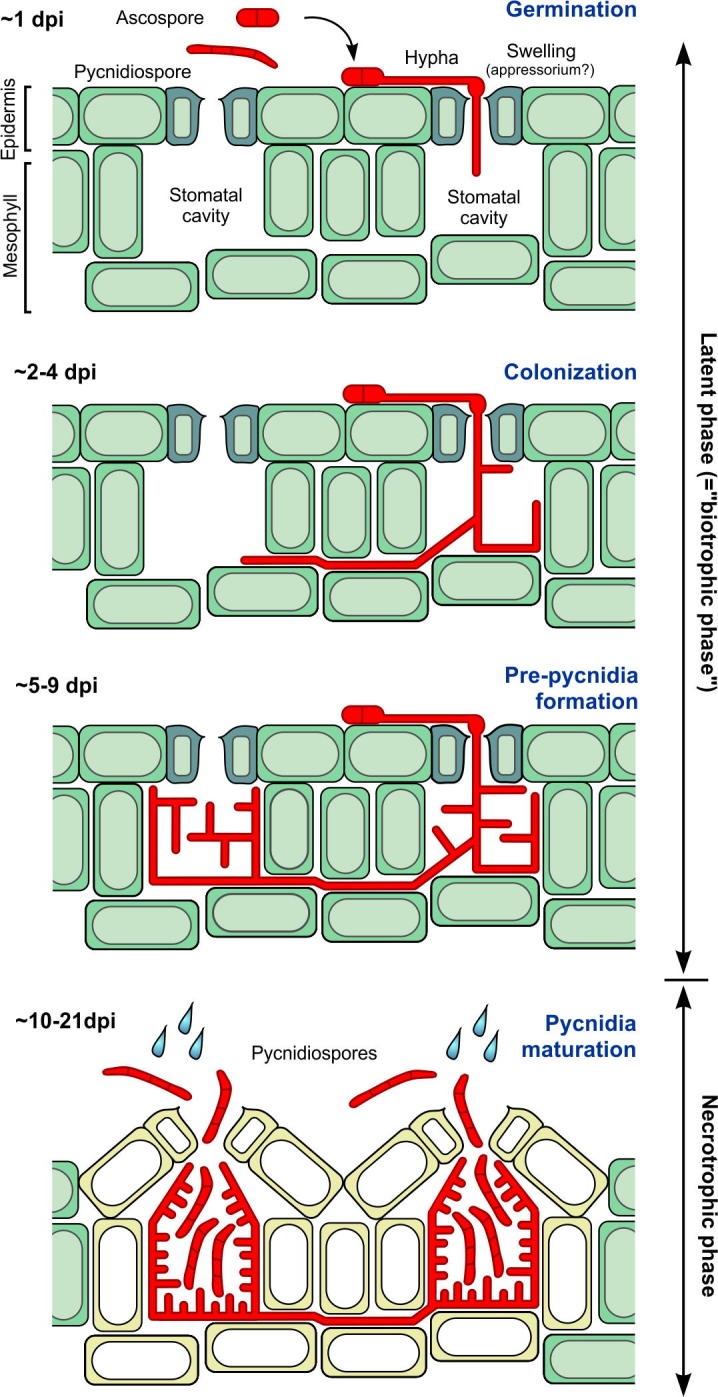
Summary of plant infection stages of *Z. tritici*. Ascospores or pycnidiospores land on the wheat leaf epidermis, where they germinate at the poles of their multi-cellular structures (Germination). Hyphae enter the leaf tissue via stomata and begin to colonize the substomatal cavity, followed by hyphal invasion of the apoplast of surrounding tissue (Colonization). Subsequently, pre-pycnidia are formed in the colonized substomatal cavities (Pre-pycnidia formation). This marks the transition from a latent biotrophic phase to the necrotrophic phase, when plant cells undergo programmed cell death. The release of plant nutrient from the dying host tissue allows rapid fungal growth and proliferation. Finally, fungal pre-pycnidia mature into pycnidia (pycnidia maturation), which produce multi-cellular pycnidiospores that are released by water splash and able to spread the infection. Note that the diagram merges several studies (Cohen and Eyal, 1993; Duncan and Howard, 2000; Kema et al., 1996a; Shetty et al., 2003), which slightly differ in details and timing of infection. Note also that pseudothecia and formation sexual ascospores is not shown.

While the importance of hyphal growth for *Z. tritici* infection is recognized, reports describing the initial steps of the infection process are not entirely consistent. Undoubtedly, *Z. tritici* hyphae enter the host tissue via substomatal openings (Fig. 3; Hilu and Bever, 1957; Cohen and Eyal, 1993; Kema et al., 1996a; Shetty et al., 2003). However, several reports describe the potential of the fungus to invade the epidermis directly, usually at anticlinal walls (Weber, 1922, reviewed in Hilu and Bever (1957), Duncan and Howard (2000), Shetty et al. (2003)). These attempts were reported to be mostly unsuccessful, or were not even found in other studies (Kema et al., 1996a), suggesting that they may reflect specialized experimental conditions, strain variations or occur in wounded tissues. Another debatable point is the existence of a stimulus that attracts hyphae to the stomata. Several studies suggested that invasion of stomata is a random process (Hilu and Bever, 1957; Kema et al., 1996a; Shetty et al., 2003). However, Howard and Duncan reported directed growth of hyphae toward the stomata, and suggested that hyphae respond to an unknown “thigmotropic signal” (Duncan and Howard, 2000). Surface sensing and “guidance mechanisms” has previously been reported in the rust fungus *Uromyces appendiculatus* (Hoch et al., 1987). Functional characterization of the protein ZtFus3 (named previously MgFus3; Cousin et al., 2006) supports the view that stomata recognition is a non-stochastic process. Deletion strains lacking this MAP kinase cannot recognize the substomatal opening, and, consequently, fail to infect the plant (Cousin et al., 2006). Interestingly, early infection in the rice blast fungus *Magnaporthe oryzae* also depends on a Fus3-like MAP kinase, PMK1 (Xu and Hamer, 1996). In this fungus, PMK1 is required for the formation of appressoria, which are specialized cells that exert mechanical pressure to force their way into the host (Deising et al., 2000). Several morphological studies report appressorium-like swellings at the tip of invading *Z. tritici* hyphae (Cohen and Eyal, 1993; Duncan and Howard, 2000; Kema et al., 1996a; Fig. 3). However, the *Z. tritici* genome lacks many of the repertoire of genes, known to be involved in appressorium formation (Goodwin et al., 2011). This suggests that *Z. tritici* does not elaborate appressoria (Cohen and Eyal, 1993). Thus, the process by which ZtFus3 enables the fungus to locate stomata and thus support early invasion remains elusive.

Fungal hyphae appear after 12–24 h in the substomatal cavity (Hilu and Bever, 1957; Shipton et al., 1971; Kema et al., 1996a; Duncan and Howard, 2000), from where they colonize the mesophyll tissue of the plant. The majority of the hyphae grow lengthwise of the leaf (Hilu and Bever, 1957), and hyphae remain exclusively in the intercellular space of the plant tissue (Hilu and Bever, 1957; Kema et al., 1996a). Within the first ∼9–15 days, they form a branched network that colonizes the plant mesophyll (Shetty et al., 2003). About 3–11 days after infection, hyphae begin to fill the substomatal space and pre-pycnidia appear in these cavities (Cohen and Eyal, 1993; Duncan and Howard, 2000; Kema et al., 1996a; Shetty et al., 2003; Fig. 3). During this course of leaf colonization, the infection remains asymptomatic. The leaves appear healthy and dead plant cells are rarely found (Hilu and Bever, 1957). This symptom-less “latent phase” (also named biotrophic phase) is unusually extended, and varies between 6 and 36 days (Shipton et al., 1971), depending on wheat genotype-fungal isolate combination (Lee et al., 2014) and, in the field, upon weather conditions. However, under laboratory conditions, it lasts usually 9–14 days (Hilu and Bever, 1957; Kema et al., 1996b; Keon et al., 2007; Shetty et al., 2007). *Z. tritici* does not form haustoria or cruder feeding structures (Shipton et al., 1971; Kema et al., 1996a). In fact, it is not entirely clear if the fungus lives on plant resources at this stage (Sanchez-Vallet, 2015). The fungal genome carries an expanded number of putative peptidases and alpha amylases, and it was suggested that the fungus metabolizes apoplastic proteins or starch, released from the chloroplasts (Goodwin et al., 2011). Alternatively, *Z. tritici* hyphae could access apoplastic sugars, which can reach high concentrations in grass leaves (Tetlow and Farrar, 1993). However, no changes in apoplastic nutrient composition were found in apoplastic metabolite analysis (Keon et al., 2007). Moreover, the fungal biomass barely increases during the initial days of infection (Keon et al., 2007; Shetty et al., 2007), which suggests that the pathogen utilizes internal stores, such as neutral lipids. It was shown that the establishment of this latent phase requires a LysM-motif containing effector protein Zt3LysM (Lee et al., 2014; Marshall et al., 2011; previously published as Mg3LysM). Such effectors compete with defence-inducing chitin receptors of the plant for binding to chitin, released from the fungal cell wall (Mentlak et al., 2012; Sanchez-Vallet et al., 2013). Indeed, such activity was described for Zt3LysM, but the protein also protects the fungal cell wall from plant-derived hydrolytic enzymes (Lee et al., 2014; Marshall et al., 2011). It was shown that plant colonization by the fungus requires the MAP kinase ZtSlt2 (previously published as MgSlt2; Mehrabi et al., 2006a). Deletion mutants were able to penetrate normally and appeared in the substomatal cavities. However, they remained un-branched and did not progress to invade the mesophyll tissue (Mehrabi et al., 2006a). A similar phenotype was observed after deletion of *ZtSte12* (previously published as MgSte12; Kramer et al., 2009)*,* suggesting that this transcription factor could function downstream of ZtStl2. One possibility is that, in the absence of this pathway, effector secretion is impaired and the fungus is unable to establish the latent phase. Future studies are needed to address this question.

Pycnidia formation is initiated in substomatal cavities (Kema et al., 1996a; Shetty et al., 2003). They are formed by extensive growth, branching and extensive fusion of hyphae, which derive originally from 1 or 2 hyphae that invaded the substomatal cavity (Hilu and Bever, 1957: Fig. 3). This colonization and the formation of pre-pycnidia occurs at 5–9 days *post* infection (Hilu and Bever, 1957; Kema et al., 1996a; Duncan and Howard, 2000). As these asexual fruiting bodies develop, chlorotic lesions appear, usually followed by necrotic areas that appear at ∼10–12 days *post* infection (Duncan and Howard, 2000; Kema et al., 1996a). This onset of the necrotrophic phase is characterized by disintegration of host tissues (Kema et al., 1996a), and this may be a consequence of extensive programmed plant cell death (Keon et al., 2007; Rudd et al., 2008). By this time, fungal hyphae have enlarged significantly (from initially 1 μm to 2.5 μm in diameter), show irregular septation and extended vacuolation (Hilu and Bever, 1957). How the necrotrophic phase is initiated is not entirely clear, but plant cell death begins in the vicinity of the substomatal cavity (Shipton et al., 1971; Fig. 3), where most fungal material in concentrated. This is consistent with the idea that the fungus secretes toxin proteins that are recognized by the plant and this hyper-activates host defenses (Kema et al., 1996a; Keon et al., 2007). Not much is known about such secreted necrosis factors, but candidates could be “necrosis and ethylene-inducing peptide 1” (Nep1)-like proteins (NLPs) – these secreted virulence factors induce necrotic cell death in several host–pathogen interactions (Gijzen and Nürnberger, 2006). The genome of *Z. tritici* contains a single NLP encoding gene, which induces necrosis in *Arabidopsis thaliana* (Motteram et al., 2009). Whilst this protein is highly expressed at the transition from the latent phase to the necrotrophic phase, deletion of the gene did not affect fungal virulence. This suggests that other, as yet unidentified, effectors trigger hyper-active plant defenses. Interestingly, genes encoding plant cell-wall degrading enzymes are highly expressed at the onset of the necrotrophic phase (Kema et al., 2008). This suggests that the fungus directly attacks the host cells, although the genome encodes surprisingly few plant cell wall-degrading enzymes (Goodwin et al., 2011). Thus, the role of these enzymes in establishing the necrotrophic phase remains elusive. In any case, the release of plant nutrients, such as amino acids and sugars, is thought to provide the basis of massive fungal proliferation and increased biomass, found during the necrotrophic phase (Keon et al., 2007; Shetty et al., 2007). Finally, mature pycnidia are formed, which produce the multi-cellular macropycnidiospores that develop from conidiophores (Hilu and Bever, 1957; Kema et al., 1996a). They are disseminated through the leaf canopy and to other plants by rain-splash (Suffert et al., 2011, Fig. 3). Asexual fruiting body development seems to be controlled by the MAP kinase ZtFus3, as deletion mutants fail to form pycnidia *in vitro* (Cousin et al., 2006). In addition, mutants in the catalytic and regulatory subunit of protein kinase A (ZtTpk2 and ZtBcy1, respectively; previously published as MgTpk2 and MgBcy1, Mehrabi and Kema, 2006) are required for pycnidia formation *in planta*. This suggests that several signaling pathways contribute to this developmental step.

In summary, the course of infection can be divided in several steps. Firstly, spores germinate on the epidermis and the fungus enters via stomata. In a second phase, the fungus shields itself from the plant recognition and colonizes the plant mesophyll. During this latent phase, changes in fungal biomass are barely detectable, most likely due to a lack of nutrients. However, fungal hyphae ramify through the leaf tissue and begin colonize the substomatal cavities around the infection site to initiate the formation of numerous pycnidia. Indeed, most recent experimental results, using highly diluted cell suspensions for infection, suggest that a single entry of a hypha is sufficient to induce the formation of large leaf lesions (Gurr and Fones, 2015). This supports the notion that extensive invasive growth occurs during the latent phase. During the third phase, the fungus strikes and initiates the necrotrophic phase, characterized by collapse of the host cells. This provides the nutrients to increase fungal biomass, leading to massive proliferation of pycnidiospores. The fourth phase is the release of spores from these fruiting bodies, which enables infection of adjacent plants. It emerges that secreted proteins may have a key role in establishing the latent phases and, probably, in initiating the necrotrophic phase (Motteram et al., 2009; Lee et al., 2014; Marshall et al., 2011). Currently, we know relatively little about these effectors. Bioinformatic approaches have revealed numerous potential candidates for secreted proteins involved in host–pathogen interaction (Morais do Amaral et al., 2012; Gohari et al., 2015). Investigating their role during infection will help understanding the cell biology of the wheat-*Z. tritici* relationship.

## 4 Perspective: How can cell biology inform disease control strategies?

In all microbes, the ability to infect the host and cause disease symptoms resides, ultimately, at the cellular level. Consequently, cell biology becomes increasingly important in modern plant pathology. For decades, cell biological research has emphasized the identification of molecules and their interactions. However, profound insight into the dynamic behavior of cells is required to “understand the causes of disease well enough to predict risks, make early diagnoses, and treat effectively” (Pollard, 2013). It was suggested that a successful strategy toward a mechanistic understanding follows three consecutive steps: (1) framing a good question, (2) identification of the molecular inventory for the process, and (3) characterization of the function of each molecule to explain its participation in a dynamic system (Pollard, 2013). In *Z. tritici* research, we are at the transition from step 1 to step 2. The pathogenic nature of *Z. tritici* dictates important questions, such as “how is the transition between biotrophic and necrotrophic phase triggered”; “how is morphological transition from yeast-like growth to hyphal growth controlled during early plant infection” or “how does the fungus establish the biotrophic phase in a compatible interaction with wheat”? In addressing these, our research has reached phase 2. Transcriptional profiling at various stages of pathogenic development (Keon et al., 2007; Kellner et al., 2014; Kema et al., 2008) and bioinformatic prediction of secreted proteins and effectors (Morais do Amaral et al., 2012; Stergiopoulos et al., 2012; Stukenbrock et al., 2011) reveals candidate genes, involved in a particular pathogenicity-relevant process. However, phase 3 requires an in-depth functional understanding of individual proteins. Targeted gene deletions have provided insight into the regulation of several steps of the infection process (overview in Orton et al., 2011). Furthermore, first putative effectors have been identified and their importance in pathogenicity of *Z. tritici* was assessed (Gohari et al., 2015; Lee et al., 2014; Marshall et al., 2011). However, we need cell biological methods, including detailed location studies, to further characterize such identified candidates. A good example, to illustrate this point, is the precise timing of effector protein secretion. Studies in the corn smut fungus *U. maydis* (Doehlemann et al., 2009; Djamei and Kahmann, 2012; Bielska et al., 2014a) and in the rice blast fungus *M. oryzae* (Khang et al., 2010; Dagdas et al., 2012; Mentlak et al., 2012; Ryder et al., 2013; Giraldo et al., 2013) provided detailed cell biological insight into infection structures and invasion strategies of these plant pathogens, using fluorescent dual labelling techniques, and revealed valuable insight into the infection process. Similar studies need to be undertaken in the wheat-*Z. tritici* system.

Surprisingly little is known when it comes to cellular organization and dynamic processes in *Z. tritici* cells. Early studies provide details on dimension and number of septa in spores (Sanderson et al., 1985; Wiese, 1987), and more recent work defines the molecular role of signaling pathways in the switch from “yeast-like” growth to hyphal growth (Mehrabi et al., 2006a,b, 2009). However, dynamic cellular pathways, such as membrane trafficking or cytoskeletal organization, are virtually not addressed. How can knowledge about fundamental cellular processes in the pathogens cell biology inform strategies toward pathogen control? As fungi adapt to existing fungicides, cellular processes need to be identified that could provide new anti-fungal targets. A good example is the identification of motile early endosome in the corn smut fungus *U. maydis*. The process of fungal early endosome motility was discovered in 2000 (Wedlich-Söldner et al., 2000). Subsequent research led to an understanding of the molecular transport machinery (Bielska et al., 2014b; Lenz et al., 2006; Schuster et al., 2011; Wedlich-Söldner et al., 2002; reviewed in Steinberg (2014)). It was shown, most recently, that early endosome motility is essential for long-range signaling during infection, which is required for effector production and successful plant infection (Bielska et al., 2014a). Thus, 14 years after its identification, fundamental research on motile early endosomes has revealed unexpected and important insights into the strategy of host invasion in *U. maydis*. While it remains to be seen if this process supports infection in *Z. tritici*, this example illustrates the principle point. We need to understand the invasion strategy and essential cell biology of the fungus to develop new ways of defence against the pathogen. Together with existing strength in population biology, biochemistry and molecular plant pathology, further discoveries in the fundamental cell biology of *Z. tritici* promise to help defeating STB.
